# 

**Published:** 1912-06-22

**Authors:** 


### OUR BUREAU OF INFORMATION.

### Rules for Correspondents.

1. Every letter, on whatever subject, must be accompanied by the coupon to be cut from the back cover (inside page) of THE HOSPITAL, current issue, and must contain the name and address of the correspondent with pseudonym for publication if desired.

2. Replies by post cannot be given, save under exceptional circumstances, at the Editor's discretion.

3. Letters from Approved Homes in reply to special needs published in the Bureau should state terms and full particulars, and be sent prepaid under cover to the Editor of the Bureau with coupon and name enclosed for identification.

4. Proprietors of homes which have not yet been entered on the List of Approved Homes, but have spare accommodation likely to suit special needs, are invited to write for an application form for registration. The fee for registration is 5s.

5. Special needs of every description relating to those who are sick or in difficulties are made known in these columns free of charge.

6. All communications to be addressed to The Editor of THE HOSPITAL, 28 Southampton Street, Strand, London, W.C., and marked "Bureau of Information."

### Homes Wanted.

### Home for Patient in Last Stages.

"J. A. D.," Bromley.—It is deplorable that the consumptive patient should be sleeping in the same room with her little child. From what you say we gather that she is too ill to be nursed in an open-air shelter in the garden, which is sometimes a good expedient when it is desired to isolate cases without sending them away from home. Write to the Lady Superintendent, The Firs Home, Bournemouth. This is a home for advanced cases of the disease, and the charge is only 10s. 6d. a week. There is also St. Catherine's Home, Ventnor, for advanced cases, where a similar charge is made. Write to Sister Bernardine. Both these homes are well managed on modern lines, with the best nursing and medical treatment.

### Home for Aged Invalid Lady.

"WEBSTER" (110A).—We note you require a nursing home in the neighbourhood of London for a lady of eighty-two recovering from an operation for cancer. The terms you name (25s.) are very low, but it is possible that one of our readers may be able to offer the required accommodation. We fear it must be considered a chronic case and should think that a permanent home where care could be given would be best. Try the Woodside Home, Whetstone, N., where there is a staff of nurses and separate accommodation for gentlewomen. Terms from £7 to £20 per quarter.

### Home for Nurse with Part Payment.

"D. E. M.," Yorkshire (109A).—We are glad to know that the Bureau has been of service in procuring you this comfortable change of air, and that you feel able to undertake light work. We are pleased to make it known that you are looking for a comfortable home in return for 10s. a week and light services. Write first under cover of Bureau to "H. O. B." (102 X), who is offering just what you require. And we will forward letters from any other correspondent able to offer you this accommodation. Write to the Lady Superintendent, 36 Aubert Park, Highbury, for particulars of home there, which might suit you.

### After-Care Home in Ireland.

MRS. L. MULLAGHMAST (111A).—We note you want a home in Ireland for a lady recovering from mental trouble where she could have some supervision before removal to her own home. We are afraid there is no home for this class of case in Ireland. Write and let us know what terms can be paid, and meantime we will gladly forward letters from any correspondent with suggestions to offer. Have you asked at the Nurses' Home and Bureau, 86 Lower Leeson Street, Dublin?

### Massage and Medical Gymnastics.

### Opening Wanted.

ENGLISHWOMAN holding high certificates from Stockholm and Paris for Swedish massage, medical gymnastics, and electrical treatment, wishes to co-operate with nursing home in London or obtain work under a physician or surgeon, providing her own costly electrical apparatus and appliances, which could be removed and re-installed if desired in other premises. We have evidence that the above is a really valuable worker, merely needing an introduction to succeed. An eminent physician writes to us that the lady in question "has had the highest possible Swedish and French training and practice in gymnastic, mechanical, and electrical therapeutic methods for several years." Prepaid replies to M. M. (Q 1) forwarded.

### Medical Queries.

### The Nature of Morgan's Bacilli.

NURSE WILLIAMS.—This bacillus is one associated with certain forms of diarrhœa, especially in children; it resembles somewhat the typhoid bacillus and belongs to that group of motile bacilli.

### Addresses Wanted.

### The Nightingale Annuities Fund.

"E. E. M.," Woking.—Write to the Secretary of above Fund, 73 Cheapside, London, E.C.

### A Centre Giving First-Aid Lectures.

THE REV. B. REES.—You would do well to communicate with Frank Hastings, Esq., Secretary of the British Red Cross Society, 9 Victoria Street, London, S.W. The London County Council, The Embankment, London, has various centres where instruction is being given in home nursing. Write for particulars.

### Hospital Appliances.

### Potato Peeler for Hand-Power.

"PENURIOUS."—A cheap and quite reliable make of machine for peeling 5 lb. of potatoes per minute by hand-power is on the market, at a cost of £3. One, indeed, was illustrated and described in the advertisement columns of THE HOSPITAL of June 1.

### Letter-Box.

DR. R.—*Re* home for maternity case (106A) in our issue of the 8th inst., we are glad to know that the Approved Home we suggested has proved suitable.

NURSE CASTLE.—See answer above to Dr. R. If you would like to have your name added to the list, write for a form of application.

"V. M."—You do not mention the age of your old servant nor what her previous work has been. What means does she possess, if any? We can advise you only if we have full particulars, and it would be well to find out her place of birth and her father's occupation, as there are many charities reserved for persons connected with certain trades or born in certain localities.

MISS HOLMES, Finchley.—We are forwarding particulars of your home to No. 108A. Please another time write a letter we could forward direct, as this saves trouble.

### Gifts and Bequests.

Mr. Frederick Ingle, of Grantham, Nottingham, and of Queen Anne's Gate, London, contractor, has bequeathed £35,000 for such charitable institutions or objects and in such proportions as his executor, in his sole discretion, may see fit, provided that not one charity shall receive more than £1,000 from his bequest and that £15,000 shall be allotted to London or national charities, £10,000 to charities in or connected with the town or county of Nottingham, and £10,000 for charities in the county of Lincoln.

### THE EDITOR'S LETTER-BOX.

### St. Bartholomew's Hospital and its Nurses.

LORD SANDHURST'S STATEMENT.

*To the Editor of* THE HOSPITAL.

### The Biennial Health Exhibition.

WHAT TO SEE.

ON Monday, June 24, and the three following days, the Biennial Health Conference and Exhibition is being held at the Royal Horticultural Hall, Westminster. It will open at 12 noon on Monday. Among the exhibits most likely to interest superintendents, stewards, and institutional workers generally are certain hygienic methods of ventilation and some water-softening appliances. Housekeepers will note the model larder, and sanatoria officials should not miss the open-air shelters and the phthisis maps which have been specially lent for the Exhibition. Hospital secretaries, in particular, will be interested in a new type of invalid lifter which Mr. Skeffington, of 49 Ulundi Road, Blackheath, has designed. The lifter in question is light and portable, stands on its own feet, and fastens to any bed. Its lightness and portability are its improved features. The "Skeffington Inclinator" for appendicitis and maternity cases is also on view, together with the patent cushion lifter for bed-pan, and a newly patented mobile sacrum lifter on wheels, which enables one nurse to do work for which two men are generally required. The Skeffington "Dysentery Mattress" also should not be missed, as its collapsible section demands a personal inspection from all who realise the peculiar strain on nurses which such cases ordinarily involve. Medical men also will be able to see for themselves specimens of Robinson's "patent" barley, which is offered as the most economical and satisfactory diluent of milk for rearing babies by hand. Crèche superintendents especially will be interested in this product, of which full information can be obtained at the Exhibition.

### Appointments.

DR. J. E. LINNELL, M.B., B.C., D.P.H., has been appointed medical officer of health to Erpingham Rural District, to which he has been acting as deputy. Dr. Linnell graduated at Cambridge University and studied at the London Hospital. His previous appointments include that of house surgeon to the London Hospital.

MR. THOMAS BEATON, M.B., B.S. (London), has been appointed sixth assistant medical officer at Long Grove Mental Hospital. He graduated at London University and has been house surgeon at Charing Cross Hospital.

COLONEL R. J. COOPER has been appointed a member of the Mental Hospitals Committee of the London County Council.

DR. HECTOR W. G. MACKENZIE, M.D., F.R.C.P., has been appointed honorary physician to the Samaritan Free Hospital for Women, London. Dr. Mackenzie is physician to St. Thomas's and to the Brompton Hospital.

DR. C. F. WALKER, B.A., assistant medical officer of health for Leicester, has been appointed medical superintendent of the Westmorland Consumption Sanatorium. Dr. Walker was educated at Queen's University, Belfast, and St. Mary's Hospital, London. He holds the degrees of M.D. in State Medicine and of B.S. of the University of London, together with the D.P.H. of Manchester University. Among his previous appointments are those of senior resident at the South Devon and East Cornwall General Hospital, Plymouth, and of resident medical officer at the Leicester Borough Sanatorium and Isolation Hospital.

ON the resignation of Dr. Hugh T. Ashby, M.R.C.P., who has been appointed an honorary physician to Salford Royal Hospital, Dr. W. A. B. Young (first assistant medical officer) has been appointed medical officer to the Out-Patients' Department of the Manchester Children's Hospital.

PROPOSED COTTAGE HOSPITAL, HAINES.—For many years in Haines it has been felt that a cottage hospital would benefit the town, and a sum of £400 was contributed in small subscriptions some time ago and still lies idle. The late Mrs. James Harris has left £2,000 for the same purpose, provided the building is commenced within two years. The chairman of the local council is Mr. Foxwarner.

